# Development of a standardized method to evaluate the protective efficiency of cosmetic packaging against microbial contamination

**DOI:** 10.1186/s13568-020-01016-4

**Published:** 2020-04-24

**Authors:** Chloe Catovic, Sylvia Martin, Stéphane Desaint, Christine Borges, Hélène Lesouhaitier, Florence Roullet, Nadine Bresciani, Anne-Marie Jouault, Valérie Poulet, Joelle Luc, Valérie Joulia, Alain Jupin, Christophe Masson, Alain Crozier, Marc G. J. Feuilloley, the members of the Cosmetic Valley EPMP commission

**Affiliations:** 1 Laboratory of Microbiology Signals and Microenvironment (LMSM EA 4312), Normandie Univ, Univ. Rouen, 55 rue Saint-Germain, 27000 Evreux, France; 2 Shiseido, 45140 Ormes, France; 3 Laboratoires de Biologie Végétale Yves Rocher, Innovation & Développement, 92130 Issy les Moulineaux, France; 4 RPC Promens, 01100 Bellignat, France; 5 Johnson & Johnson Santé Beauté France, Val de Reuil, 27100 France; 6 Aptar Beauty & Home, 27380 Charleval, France; 7 Chanel Parfum Beauté, 93694 Pantin, France; 8 Sisley, 95310 Saint Ouen L’Aumone, France; 9 Clarins, 95300 Pontoise, France; 10 Laboratoires Pierre Fabre Dermo Cosmétique, 31322 Castanet Tolosan, France; 11 Albéa, 92635 Gennevilliers, France; 12 Cosmetic Valley, 28000 Chartres, France; 13 Clean Cosmetic Consulting, 77420 Champs Sur Marne, France

**Keywords:** Cosmetic packaging, Microbial contamination, Quality, Evaluation

## Abstract

Doubts surrounding the potential adverse effects of antimicrobial preservatives have modified the demand of consumers, who increasingly insist on the production of low-level and even preservative-free cosmetics. Protection of the product against microbial contamination is therefore focused on the packaging. This has prompted the emergence of a highly diverse array of so-called “protective”, “overprotective”, and “barrier” packaging. However, these designations are not normalized and the choice of the right packaging adapted to each cosmetic product is still essentially empirical, hazardous, and time consuming. The Cosmetic Valleys cluster has launched a commission to define a complete and experimentally-validated method to classify the level of protection of cosmetic packaging against microbial contamination. As reported herein, this required the development a specific bacteriostatic medium that can be used for 7 days and an in vitro procedure that reproduces in-use contamination and consumer practices. Based on tests performed on over 800 packages of different origin and performance characteristics, we propose a classification, divided into six grades, to differentiate the protective efficiency of cosmetic packaging. This work can be considered as a first step towards a regulatory text.

## Key points

Evaluation of the adaptation of cosmetic packaging to protect the product against microbial contamination.

## Introduction

Demonstration of the health and environmental risks associated with the presence of preservatives in personal-care products and cosmetics has prompted the emergence of low-level and even preservative-free products (Halla et al. 2018). As such formulations are particularly sensitive to microbial contamination, protection of the product has focused on the packaging. The role of packaging in the preservation of cosmetics is well known and the packaging itself is considered to be an essential part of the final product in regulatory texts (Lundov et al. 2009; Regulation EC 1223/2009 protection of the product has been developed (Crozier 2018a). Such packaging has essentially two functions, protecting the product in the device by limiting all potential retro-contamination and ensuring delivery of minimally-contaminated and safe doses throughout the shelf life of the product. These objectives are somewhat contradictory, as it is particularly difficult to associate increased hermetic closure and regular delivery of the content throughout the life of the product, except for single use devices, which are faced with other problems, such as increased contain-container interactions (Feuilloley and Orange 2018) and ecological compatibility (Thompson et al. 2009). In addition, delivery of a dose implies the potential accumulation of remaining product inside (dead space zones) and/or even outside of the device, with a high risk of contamination when the product is not self-protected.

As the packaging is an intrinsic element of low-level and preservative-free cosmetic products, it is essential to adapt its performance to the protection of the formulation to insure sanitary safety. Conversely, as certain active cosmetic ingredients also have antimicrobial activity, even in the absence of added preservatives (Papageorgiou et al. 2010; Herman et al. 2013), the formula can provide a certain amount of protection against microbial contamination and the pack should be adapted to this situation to avoid over-quality costs. Protocols have been proposed to evaluate the microbial protection provided by cosmetic packaging (Devlieghere et al. 2015; Briasco et al. 2016). However, the packaging and formulation are generally produced by different partners and, in the absence of regulatory texts and even technical tools that make it possible to define a clear hierarchy from low to overprotective and barrier packaging, selecting the correct association is essentially empirical and potentially hazardous.

The Cosmetic Valley cluster, the world’s leading center for resources in perfumery and cosmetics, offers the possibility to associate professionals of the packaging and cosmetic industries with academic investigators to define, for the first time, a technically functional harmonized procedure that could be applicable to all industrial partners and potentially translated into a future harmonized regulatory standard. Here, we describe the logical and technical approach used to define such a procedure, the experimental studies conducted to overcome fundamental technical obstacles, and the tests performed for its validation.

## Materials and methods

### Definition of the target and regulatory context

A “Guideline for the evaluation of physical antimicrobial protection provided by packaging” was developed for the comparison of the performance of commercially available cosmetic packaging, taking into account the physical properties (fluidity) of the potential final formulation and, independently, its composition. This procedure can also be used to compare devices under development, although in this case, the true level of protection would remain relative. The results should be integrated into a microbial risk assessment procedure, as described by ISO 11930 (2019) and ISO 29621 (2017). It is essential that it be possible for the tests to be performed by the packaging and cosmetic industries. Thus, as the staff is normally trained to work under sterile conditions and has access to the necessary equipment, the biological risk of all microbial strains had to be level two or lower (European Community classification 2005). One of the goals was to also take into account the true practices of the consumer, as they are the principal source of contamination. Thus, a specific in vitro contamination procedure was developed to mimic normal use.

### Selected bacterial model

To be economically realistic, it became rapidly apparent that the procedure should provide results using a single model microorganism. In addition to being level two or below, the following key criteria were retained for its selection:

- Presence in the microbial library of most industrial sites.
- Aerobic and easy to grow.
- Average size to avoid potential under estimation of the protective performance of the packaging.
- Frequently found in the environment and potentially on human skin.
- Mobile, so that it can diffuse throughout dead spaces and be detectable.

Considering all these elements, the species *Pseudomonas aeruginosa* was selected. All tests were realized using the strain ATCC^®^ CRM-9027. Equivalent strains CIP^®^ 82.118, NCIMB^®^ 8626, NBRC^®^ 13275, or KCTC^®^ 2513 should also be usable.

### Type of packaging and sampling procedure

Predictable low-level non-protective packaging against microbial contamination, such as pots or open tubes, which provide no protection to the contents, were excluded from the study. At the other end of the protection spectrum, hermetic, sterile, single-use packaging, corresponding to pharmaceutical-quality products, was also considered to be out of the scope of the present study.

It was thus decided to develop the present procedure for two types of devices:

- Overprotective packaging.
- Absolute barrier packaging.

Given their production mode, it was decided that the tests had to be conducted on a minimum of 50 randomly selected devices to reach reliable statistical values. Before entering into the testing procedure all devices were decontaminated using a technique adapted to their composition (ionization, autoclaving, etc.). All devices were filled under sterile conditions and control sterility tests were performed.

### Testing media

Overprotective packaging is not meant to be totally hermetic to contamination. As one of the targets of the protocol was to take into consideration the real “in use” practices of the consumer, it was decided that the contamination procedure should be repeated for at least a week. The contamination level of distributed doses can be measured immediately, but that of the formulation present in the reservoir of the device can only be measured after opening. Given that bacteria can multiply very rapidly, it would be impossible to determine at which time of the test the initial contamination occurred. Thus, it was necessary to develop a bacteriostatic medium for *P. aeruginosa*. In addition, this bacteriostatic medium had to be supplemented with a reticulation agent to adapt the viscosity, as the performance of the packaging is adapted to the mean fluidity of cosmetic creams.

Barrier packaging should normally block all bacterial contamination, while delivering repeated doses. In this respect, perfectly safe absolute barrier packaging is yet to be developed, but very high efficiency barrier devices, adapted to low protection formulae, have been marketed. Therefore, we tested such packaging using a classical fertile medium for *P. aeruginosa*. However, such packaging is also designed to be used with cosmetic creams. Thus the classical fertile media had to be formulated using a reticulation agent, as it is normally too fluid.

### Contamination procedure

**Fig. 1:**
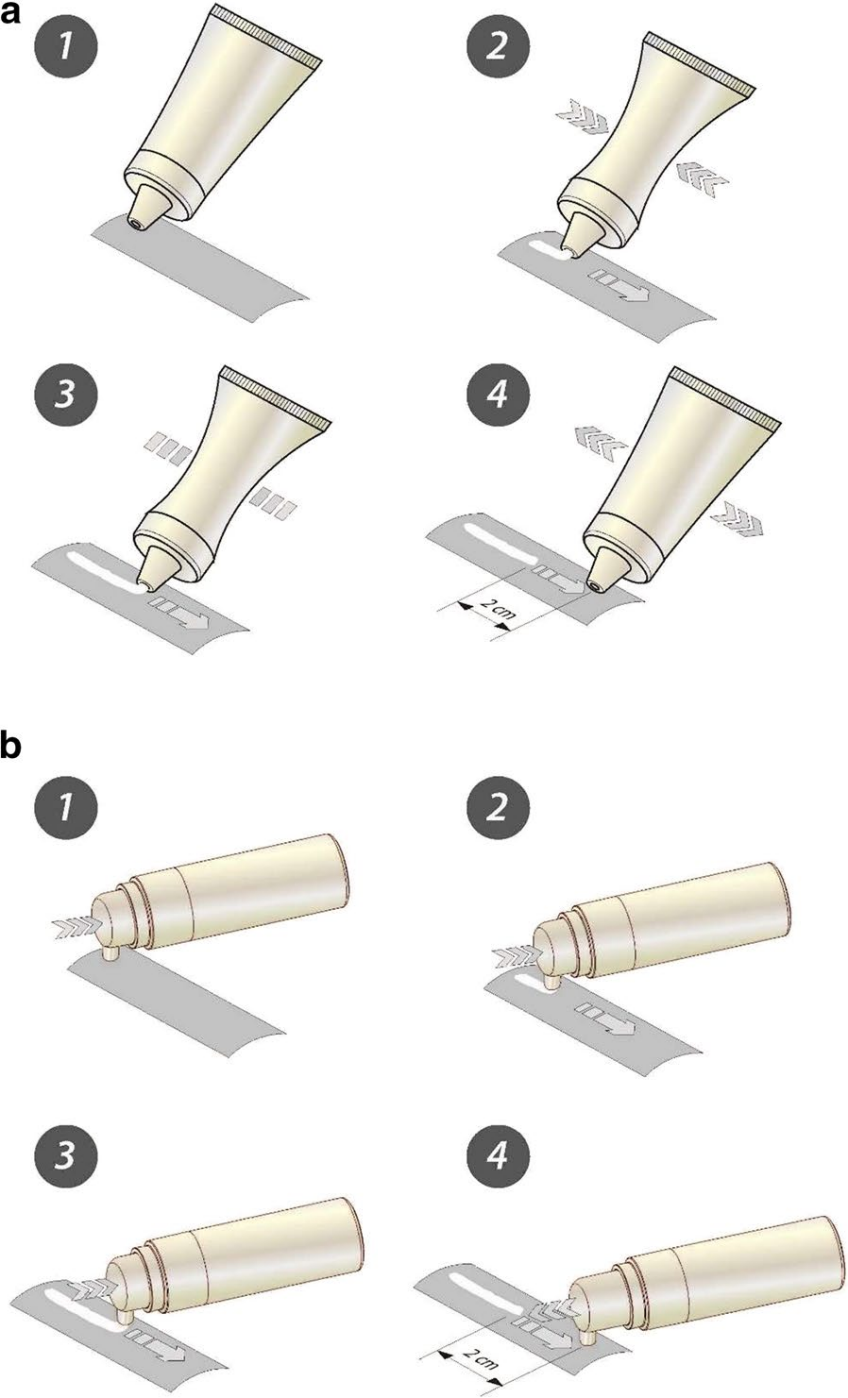
Illustration of the contamination procedure developed to reproduce natural contamination due to skin contact under standardized conditions. For flexible packaging (**a**), the nozzle of the pack is pressed in contact with the contaminated compress (10^6^ CFU/mL) (1) and the dose (as defined by the provider) is released (2). When it is completely restituted (3), the pressure on the packaging is relieved and the movement is prolonged for a distance of 2 cm (4) to mimic manual wiping of the tip. This procedure can be adapted to rigid packaging (**b**) by replacing the pressure on the side of the pack by actuation of the delivery pump

To reproduce normal use of the device, the packaging was exposed to contamination by wiping the nozzle while dispensing a dose on a sterile compress (30 g/m^2^, NF-EN 29073-1) impregnated with bacteria at a concentration of 10^6^ CFU/cm^2^ (Fig. 1). This bacterial concentration was selected to be equivalent to the mean bacterial load of human skin (Wilson 2005). The compress was open to form a 100 × 200-mm rectangle and inserted into a sterile stomacher bag to ensure homogeneous contamination. The bacterial solution (5 mL, 10^8^ CFU/mL) was distributed over the entire surface of the pad and after closure of the bag, the liquid was spread by exerting a gentle pressure from the center to the periphery. This procedure was validated by testing colored media supplemented with phenol red to verify the homogeneous distribution of the solution.

The contamination simulation was conducted over 8 days at room temperature, as presented in Table 1. Given that days 1 and 2 were used for carrying out the controls, filling the packaging, and sub-culturing the bacteria, the true simulation procedure started on day 3 by controlling the sterility of the package and exposition to the contaminated pad in the morning and afternoon at 6-h intervals (minimum). Thereafter, the packaging was exposed twice a day at 6 h intervals to the contamination from days 4 to 7, as shown in Fig. 1, and two doses were collected. A first dose was also collected on day 3 before the first daily contamination and was used as a control. On day 8, the bacteria in 1 g of delivered medium were enumerated. Then, the packaging was opened under sterile conditions and an aliquot of the formulation remaining in the container was collected and plated on Petri dishes to assess potential contamination that developed in the container.

**Table 1:** Schedule of the simulation contamination procedure

Day	1	2	3	4	5	6	7	8
Bacterial strain subculturing		Yes	Yes	Yes	Yes	Yes		
Inoculum preparation			10^6^ UFC/cm^2^ on pads	10^6^ UFC/cm^2^ on pads	10^6^ UFC/cm^2^ on pads	10^6^ UFC/cm^2^ on pads	10^6^ UFC/cm^2^ on pads	
Medium control	Numeration							
Packaging sterility control	(Filling)	Numeration	Numeration (unused pack)	Numeration (unused pack)	Numeration (unused pack)	Numeration (unused pack)	Numeration (unused pack)	
Delivered doses numeration (morning)			1 dose before simulation	1st dose 2nd dose	1st dose 2nd dose	1st dose 2nd dose	1st dose 2nd dose	Numeration on 1 g + pack content
Simulation (6 h minimum between each)			Morning	Morning	Morning	Morning	Morning	
			Afternoon	Afternoon	Afternoon	Afternoon	Afternoon	

For determination of the contamination level of the delivered doses, each dose was weighed and diluted 1/10 and 1/100 (w/v) in Eugon LT100 medium (Fisher Scientific) before plating on tryptase soya broth (TSA)-agar solid medium. Eugon LT100 was necessary to neutralize the potential remaining bacteriostatic activity of the medium used to test the overprotective packaging. CFU were counted after 48 to 72 h of incubation at 32.5 ± 2.5 °C.

## Results

### Formulation of the bacteriostatic and fertile media

There are many media available for increasing the cultivability of bacteria. Conversely, bacteriostatic media are rarely available, particularly for a versatile species such as *P. aeruginosa*. Moreover, although certain media have bacteriostatic activity lasting for 48 or even 72 h (Zwisler laboratorium^®^ medium), we required a medium that remained bacteriostatic over a minimum of seven days to avoid any artefacts in the evaluation of the packaging. Another challenge was the obligation to adapt the fluidity of the medium to be compatible with the mean fluidity of the cosmetic formulations classically employed in the packaging. All compounds also had to be of limited cost and compatible with the safety rules of industrial companies.

Starting from the composition of the Zwisler laboratorium^®^ medium, we performed a series of tests to develop a medium with bacteriostatic activity on *P. aeruginosa* that lasted for more than 1 week using media with the basic formula:

- DPBS (Dulbecco’s Phosphate-Buffered Saline) (Thermo Fisher) 900 mL.
- Glycerol (Carl Roth) 100 mL.
- MgSO_4_ (anhydrous) (Sigma Aldrich) 0.4 g.
- Phenol red (Merck Millipore) 5 mg.
- Low viscosity carboxymethyl cellulose (Sigma Aldrich) 25.0 g.
- Bacteriostatic agent (varying percentage for tested molecules).

**Fig. 2:**
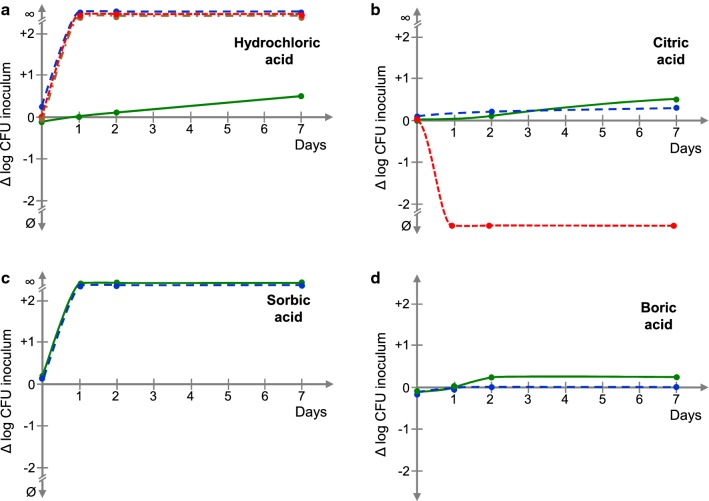
Comparison of the bacteriostatic activities of media produced in the presence of different acids. The evolution of the contamination was measured over 7 days and was expressed as the logarithmic variation of the initial inoculum (Δlog CFU: colony-forming unit). **a** Hydrochloric acid pH = 5.39 green lines, pH = 5.45 blue dotted lines, pH = 5.63 red dotted lines, and pH = 5.91 orange dotted lines. **b** Citric acid pH = 4.79 green lines, pH = 4.81 blue dotted lines, and pH = 4.94 red dotted lines. **c** Sorbic acid 0.05% green lines and 0.1%. **d** Boric acid 0.84% (pH = 5.41) and 0.26% (pH = 6.00 blue dotted lines). All experiments were performed in triplicate and completed with tests at higher and lower concentrations (not shown)

**Fig. 3:**
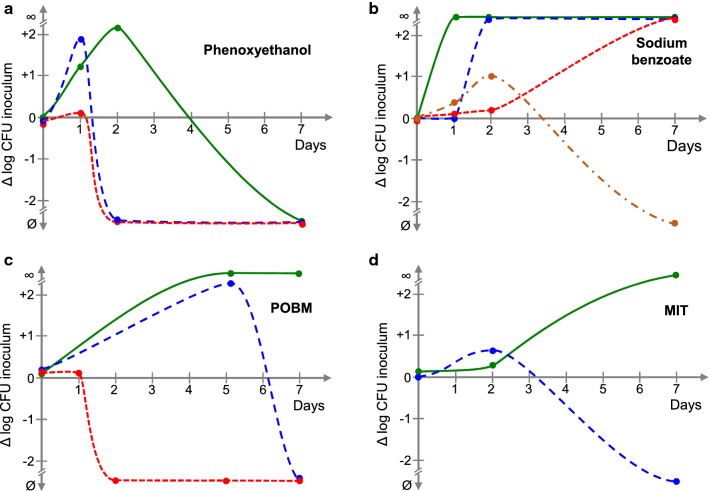
Comparison of the bacteriostatic activities of media produced in the presence of common organic preservatives. The evolution of the contamination was measured over 7 days and was expressed as the logarithmic variation of the initial inoculum (Δlog CFU: colony-forming unit). **a** Phenoxy ethanol 0.3% green lines , 0.32% blue dotted lines and 0.35 red dotted lines%. **b** Sodium benzoate 0.01% green lines, 0.03% blue dotted lines, 0.04% red dotted lines, and 0.05% orange dotted lines. **c** POBM (methyl paraben) 0.1% green lines, 0.12% blue dotted lines and 0.15% red dotted lines. **d** MIT (methyl isothizolinone) 0.001% green lines and blue dotted lines 0.002%. All experiments were performed in triplicate and completed with tests at higher and lower concentrations (not shown)

All tested media were inoculated at day 0 with 100 UFC/g of *P. aeruginosa.* For each bacteriostatic agent tested, the evolution of the contamination was measured over 7 days and was expressed as the logarithmic variation of the initial inoculum (Δlog colony-forming unit). The principal bacteriostatic agents studied are presented in Fig. 2 (acids) and in Fig. 3 (common organic preservatives). As previously mentioned, these substances were selected as they are low cost and present in most industrial companies. Hydrochloric acid (Fig. 2a), citric acid (Fig. 2b), and sorbic acid (Fig. 2c) were tested based on the hypothesis that a decrease in pH affects the growth of *P. aeruginosa* (Sporer et al. 2017). Boric acid (Fig. 2d) was tested as, in addition to its acidifying effect, borate ions can also affect bacterial growth (Lum and Meers 1989). Other common preservatives were also tested, such as phenoxyethanol (Fig. 3a) used in cosmetics for its broad preservative activity (ANSM 2012). Sodium benzoate (Fig. 3b) a food preservative also used in pharmaceutical formulations was included in the tests. Methyl isothiazolinone (MIT) (Fig. 3c), a powerful synthetic biocide, was also tested, despite its known skin sensitization activity. We also decided to test methyl paraben (methyl parahydroxybenzoate MPOB), the E218 food preservative (Fig. 3d). These compounds were studied over a wide range of doses, although only the more relevant results are shown in the figures. Caprilyl glycol, a skin conditioning agent with antimicrobial activity, and nalidixic acid, a DNAgyrase inhibitor known to block bacterial division were also tested in preliminary studies (data not shown).

Hydrochloric acid (pH = 5.39) (Fig. 2a) showed inhibitory activity, but this effect was lost with very limited increases in the pH. At pH = 5.45 and above, the contamination level of the medium at day 1 was too high to be determined (∞). Citric acid (Fig. 2b) partially blocked the growth of *P. aeruginosa* at pHs = 4.79 and 4.81 but acted as an inhibitor at pH = 4.94. Sorbic acid (Fig. 2c) was completely ineffective in blocking the growth of *P. aeruginosa* at any concentration tested (0.05 to 0.1%). Boric acid (Fig. 2d) showed good bacteriostatic activity on *P. aeruginosa*, particularly when used at 0.26% (pH = 6.00) or 0.84% (pH = 5.41). At higher concentrations boric acid was lethal to the bacteria. The response of *P. aeruginosa* to phenoxyethanol (Fig. 3a) was unexpected. Except at the highest concentrations used (0.35% and above), the bacteria grew for one or two days but then stopped, probably due to the toxic effect of this molecule. Up to a concentration of 0.045%, sodium benzoate (Fig. 3b) was only able to delay the onset of *P. aeruginosa* multiplication, and no detectable viable bacteria were detected at day 7 at higher dose (0.05%). Methyl paraben (POBM) (Fig. 3c) also showed a varying response, ranging from a limited inhibition of bacterial growth at 0.1% to a complete inhibition at 0.15%. At day 7, the effect of methyl isothizolinone (MIT) (Fig. 3d) passed from the total absence of inhibition at 0.001% to total inhibition at 0.002%. Thus, except for boric acid, all tested compounds were unable to inhibit the development of *P. aeruginosa* over 7 days or the range of their effective concentration was so narrow (particularly pH values) that their use in reproducible tests was impractical. Finally, although the use of boric acid is restricted because its classification by the EU as a carcinogen, mutagen, and reprotoxic (CMR) 1B or 2, depending on its concentration, it was selected for production of the bacteriostatic medium. However, the viscosity of the first bacteriostatic medium was too low, although it included carbomethylcellulose. Thus, a second series of tests was carried out using various reticulation agents. The viscosity of the formula was estimated visually (Table 2) and that of the more suitable solutions measured using a Viscotester IQ Haake rheoviscosimeter with coaxial cylinders and a CC25 DIN/Ti gap. Considering the range of the mean fluidity (18,000 ± 3000 mPas at 25 °C, shear 15 s^−1^) of most typical cosmetic lotions, hydroethylcellulose (HEC, 25 g/L) was selected to formulate the bacteriostatic medium. The activity of this medium was verified using a 100 CFU/mL *P. aeruginosa* inoculum, as any modification of the formula can influence the bacteriostatic properties. It was validated based on a maximal variation < 50% (0.3 log), according to standard guidelines (ISO 2114).

**Table 2:** Reticulation agents tested and apparent fluidity of the media

Reticulation agent	Reference	Viscosity
1. Gelrite	Carl Roth Ref 0039.1	Thixotropic—Formation of lumps
2. Carboxymethylcellulose (CMC) Low vicosity	Sigma Aldrich Ref C5678	High fluidity even at XX% and independently of the pH
3. Carboxymethylcellulose (CMC) High viscosity	Sigma Aldrich Ref C5013	Gel like structure
4. Hydroxyethylcellulose (HEC)	Ashland Ref Natrosol 250 HX	Gel like structure
5. Carbopol (CP) Ultrez 30 Polymer	Lubrizol Ref CBP1118	Fluid cream independently of the pH
6. Carbopol (CP) Ultrez 10 Polymer	Lubrizol Ref CBP10954	Fluid cream independently of the pH
7. Aristoflex AVC	Clariant Ref 1382402689	Fluid cream independently of the pH

As previously mentioned, selecting a fertile medium was a simpler task. It included MgSO_4_, glycerol, and phenol red at the same concentrations as those used for the bacteriostatic medium to keep the two formulations similar. DPBS was replaced by Trypcase Soja Broth (TSB) to favor bacterial growth. However, the use of TSB modified the fluidity of the medium and it was necessary to increase the amount of reticulation agent (HCE) to 25.7 g/L to preserve the same rheological properties.

### Comparative tests of overprotective and barrier packaging

A total of 16 series of 50 packages were submitted for in-use tests in duplicate by five different cosmetics producers. These tests were performed by seven different operators. They included five types of packaging of different protective levels (presumably overprotective or barrier) from three different producers. The packages were anonymized and identified by letters. They were transmitted without the origin of reference to the laboratory in charge of the tests. The results are presented in Table 3. One of the packages (A) provided a limited level of protection, as all delivered doses 1 and 2 were contaminated, generally with a high number of microorganisms (less than 30% of the delivered doses contained < 1000 UFC of contamination). Package B provided better protection, although some of the delivered doses contained > 1000 UFC of contamination. Package D gave better results, in which all delivered doses for one series of tests showed < 1000 UFC of contamination, but the performance was irregular. Packages C and E showed the best level of protection in these tests. As expected by overprotective or barrier packs, all the tested devices provided complete protection of the reservoir content.

**Table 3:** Results from in-use tests

	Results at days 4, 5, 6 and 7				Results at day 8				
Pack code	Delivered dose 1	Delivered dose 2	Delivered dose 1	Delivered dose 2	Delivered dose 1 (1 g)	Delivered dose 2 (1 g)	Reservoir		
	Non contaminated (%)	Non contaminated (%)	Contamination < 1000 UFC (%)	Contamination < 1000 UFC (%)	Non contaminated (%)	Contamination < 1000 UFC (%)	Non contaminated (%)	Contamination < 10 UFC (%)	Contamination < 1000 UFC (%)
A	0	0	5	7.5	0	0	100	100	100
	0	0	0	0	0	0	100	100	100
	0	0	2.5	30	0	0	100	100	100
B	0	0	60	70	40	90	100	100	100
	10	7.5	62.5	80	60	90	100	100	100
C	37.5	85	100	100	100	100	100	100	100
	2.5	15	70	87.5	0	100	100	100	100
	0	0	5	15	0	20	100	100	100
D	45	52.5	75	82.5	100	100	100	100	100
	0	0	12.5	22.5	0	10	100	100	100
	0	0	0	72.5	0	40	100	100	100
E	32.5	82.5	100	100	100	100	100	100	100
	0	0	22.5	75	60	100	100	100	100
	2.5	10	50	72.5	60	80	100	100	100
	0	0	42.5	90	100	100	100	100	100
	10	0	65	85	60	90	100	100	100

A total of 16 series of 50 devices was tested in duplicate (i.e. 800 packs)

Values are expressed as percentages of the number of tested packs in order to make easier the comparisons

## Discussion

Faced with the diversity of technical solutions proposed to preserve low-level and preservative-free cosmetic products, both the packaging and cosmetic industries should gain from the existence of harmonized standards. In addition to regulatory texts, several rare studies have been conducted to validate the microbial safety of cosmetic packaging (Crozier 2018a). However, this study is the first to validate an experimental protocol over a wide range of devices and associated them a classification grid allowing their differentiation based on the degree of microbiological safety conferred by the package.

*Pseudomonas aeruginosa* was selected as a model because of its listing as a challenge test microorganism (ISO 11930 2019) and therefore its presence in the microbial library of most industrial sites. In addition, this bacterium grows easily, it is of average size, is widely distributed throughout the environment, and is mobile due to its polar flagella. This bacterial model has the drawback of being a safety level 2 microorganism and thus requires specific facilities. This was preferred to a ubiquitous safety level 1 species, such as *Pseudomonas fluorescens* (Bossis et al. 2000), as this bacterium is not generally available and manipulated in industry and our aim was that our approach be applicable for packaging users and producers. The choice of *P. aeruginosa*, aside from and because of its versatility, was also probably at the origin of the difficulty to formulate a medium that preserved its bacteriostatic activity for 7 days. This can appear to be trivial, but although bactericidal and fertile medium can be easily found in the literature, this is the first time that an exhaustive study has been performed on a *P. aeruginosa* bacteriostatic medium, particularly one that functions over a long time. The efficiency of boric acid in the formulation of a bacteriostatic medium relative to all other tested substances is supported by its long-standing use as a preservative for the transport of urine samples for clinical observations (Lum and Meers 1989). Boric acid was more recently proposed for the preservation of urine samples for veterinary applications (Rowlands et al. 2011). The major limitation for the use of boric acid is its classification as hazardous (CMR2 or 1, depending on the exposure concentration) by the European Chemical Agency (ECHA European Chemical Agency 2010). Here, boric acid was used at a maximum concentration of 0.485% (mass/vol). Thus, only manipulation of the bulk material should require CMR protection protocols.

Another important aspect of the protocol concerned the development of an in-use contamination procedure aimed at reproducing the practices of the consumer. This is essential, as contact with the skin and its natural microbiota is the major source of contamination for cosmetic products. In addition, in case of incomplete closure, wiping the outlet can generate limited pressure and favor reflux of the product into the container and contamination of the reservoir. Another problem is that if a significant amount of product remains in the outlet between successive uses and the product is not self-protected, this may be sufficient to permit active bacterial growth and delivery of a contaminated dose. The contamination protocol presented in Fig. 1 and Table 1 was established to evaluate the two parameters, i.e. the microbial contamination of the delivered dose and potential contamination of the reservoir. Although it was impossible to investigate the microbiological protection of the packaging throughout its entire lifecycle, including production and storage, an in-use test that spanned 7 days appeared to be coherent with validation protocols in regulatory texts. In addition, the validation tests were conducted on a series of 50 packages to account for normal variability of the quality. Indeed, packages are not generally produced one by one but using molds with multiple forms and, in spite of controls, not all forms are strictly equivalent in performance.

**Fig. 4:**
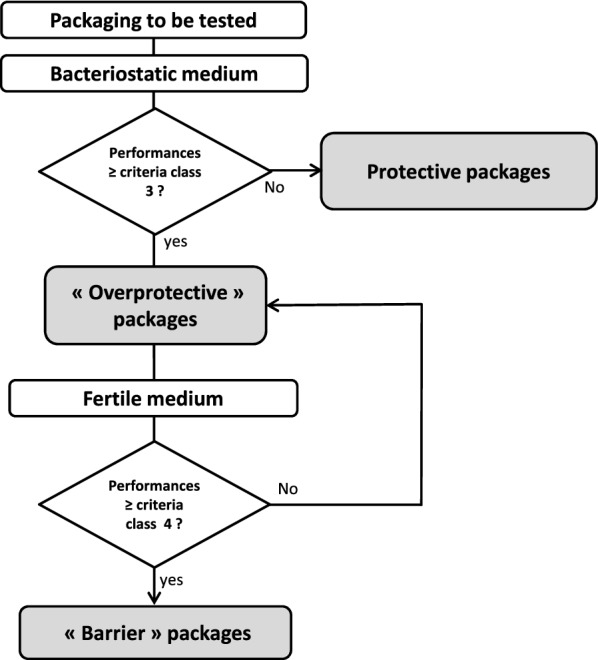
Flowchart established to classify the microbial protection potential of packaging

Given the results, we decided to classify the packaging into six categories ranging from 0, corresponding to the complete absence of protection, to 5, providing complete protection. Devices, such as overprotective and barrier packaging, that were tested in the present study should be classified between 2 and 4, depending on the percentage of contaminated doses measured during the test. In all these devices, the container was absent of contamination. Grade 3 and 4 packaging are differentiated by the tests on fertile medium, which are the most discriminant, and only level 4 can pass the tests with respect to the criteria presented in Fig. 4. As this procedure was aimed for products for public use and for use by all interested producers of packaging and cosmetics, it was decided to propose a logotype that should be printed on the identity file of the products. This logotype was deposited to the French organism for the control of intellectual property (INPI) under the property of the Cosmetic Valley cluster. Its use is free but restricted to cosmetic packaging and requires that the device meets the criteria summarized in Table 4.

**Table 4:**
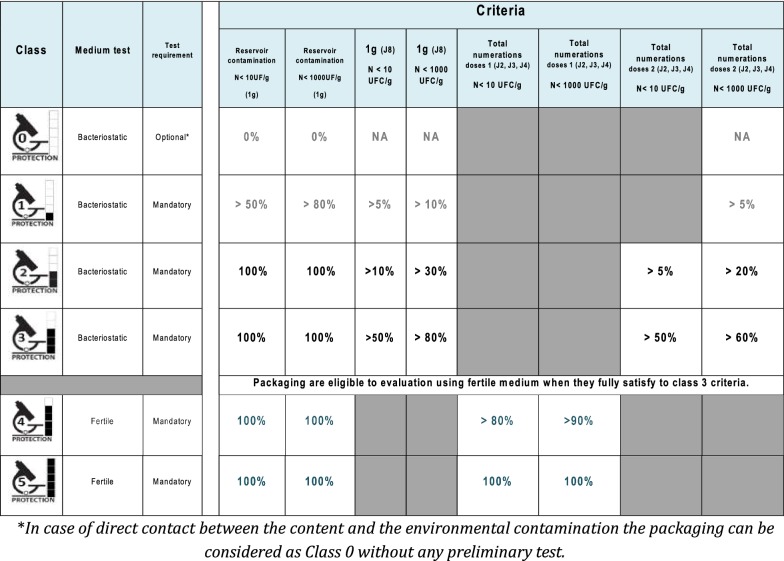
Classification of the packs in regard of their microbiological protection properties and criteria of each class established on the basis of in-use tests

In conclusion, this study, which has brought together some of the major industrial partners of the cosmetic and packaging industry over the last 5 years, has resulted in the first complete and experimentally validated protocol that allows the selection of cosmetic packaging as a function of the expected level of microbial protection. This work was presented during a congress to members of the International Organism for Standardization (Crozier 2018b; Feuilloley and Roullet 2018), who will now consider its translation into a new international regulatory text.

## Data Availability

All experimental results are available upon simple demand to the authors. Results on the packaging have been kept anonymized to avoid any commercial application.
